# Development of Low-Contact-Impedance Dry Electrodes for Electroencephalogram Signal Acquisition

**DOI:** 10.3390/s23094453

**Published:** 2023-05-02

**Authors:** Ramona B. Damalerio, Ruiqi Lim, Yuan Gao, Tan-Tan Zhang, Ming-Yuan Cheng

**Affiliations:** Institute of Microelectronics, Agency for Science, Technology and Research, Singapore 138634, Singapore

**Keywords:** dry electroencephalogram (EEG) electrodes, low-impedance electrodes, polydimethylsiloxane (PDMS), flexible printed circuit board (FPCB)

## Abstract

Dry electroencephalogram (EEG) systems have a short set-up time and require limited skin preparation. However, they tend to require strong electrode-to-skin contact. In this study, dry EEG electrodes with low contact impedance (<150 kΩ) were fabricated by partially embedding a polyimide flexible printed circuit board (FPCB) in polydimethylsiloxane and then casting them in a sensor mold with six symmetrical legs or bumps. Silver–silver chloride paste was used at the exposed tip of each leg or bump that must touch the skin. The use of an FPCB enabled the fabricated electrodes to maintain steady impedance. Two types of dry electrodes were fabricated: flat-disk electrodes for skin with limited hair and multilegged electrodes for common use and for areas with thick hair. Impedance testing was conducted with and without a custom head cap according to the standard 10–20 electrode arrangement. The experimental results indicated that the fabricated electrodes exhibited impedance values between 65 and 120 kΩ. The brain wave patterns acquired with these electrodes were comparable to those acquired using conventional wet electrodes. The fabricated EEG electrodes passed the primary skin irritation tests based on the ISO 10993-10:2010 protocol and the cytotoxicity tests based on the ISO 10993-5:2009 protocol.

## 1. Introduction

Electroencephalogram (EEG) monitoring is a noninvasive procedure that is used to record brain signals and detect abnormal brain activity. EEG recording is conducted for various reasons, such as primarily diagnosing head injuries, sleep disorders [1], seizures (such as epilepsy [2]), brain tumor and brain dysfunction, and memory problems (such as dementia [3,4]) as well as detecting brain signals in individuals in a coma [5]. Utilization of EEG systems has expanded beyond clinical settings over the last decade. Neuropsychological studies have shown correlation between emotions and EEG signals. This involved artifact filtering, EEG feature extractions, use of machine learning classifiers [6], and predictions from convolution neural network and long short-term memory [7]. Major depressive disorder is detected from graph structures formulated from EEG signals [8]. Baygin et al. utilize Collatz pattern modeling of EEG signals for automated detection of schizophrenia [9]. EEG-based rehabilitation of limb functions following a stroke has also been studied [10,11,12]. Foong et al. utilized visual feedback in EEG-based study to help stroke survivors with short-term upper limb improvement and detect possible presence of mental fatigue [13]. Moreover, commercial EEG gaming systems have been used to measure auditory event-related potentials which are correlated with cognitive disorders [14]. Negative contents in various mixed reality (MR) games are also avoided though classification of EEG signals collected while playing MR games [15]. 

Most of these recent EEG-based studies utilize dry EEG systems. Mobile, portable, and wearable dry EEG systems have been developed for use outside of clinical and laboratory settings [16]. Aside from various brain–computer interface (BCI) and machine learning classifiers, dry electrodes must be reliable in capturing brain signals in dry EEG systems. They are easy to set-up and are capable of detecting EEG signals comparable to wet systems [17,18]. EEG-based studies have enhanced not only the interaction between human and machine but the user’s engagement in purchasing intention [19]. 

In conventional EEG monitoring systems, a conductive substance, such as an electrolytic gel, a high-viscosity conductive paste is placed on flat and shallow cupped metal disks (i.e., EEG electrodes). Thus, such systems are commonly called wet systems. Scalp or skin preparation is an essential and tedious process for a wet EEG system. The EEG measurement usually follows the standard 10–20 electrode arrangement, under which electrodes are placed at a relative distance of 10% or 20% of the skull distance from each other (as proposed by the International Federation of Societies for Electroencephalography and Clinical Neurophysiology) [20]. These setup and skin preparation processes usually require a combined time of 20–30 min. Moreover, an EEG recording usually takes approximately 30–60 min, depending on the quality and duration of the EEG signals requirement. The conductive paste is used to facilitate the acquisition of stable EEG signals and reduce the electrode–skin contact impedance [21]. For ambulatory cases that require ≥24 h of recording, degradation of the brain signal or contact impedance quality is probable as the conductive substance dries out [22]. After EEG recording, patients are required to clean their head for conductive paste removal.

Dry EEG systems have a short set-up time, require limited hair preparation, and do not require skin scrubbing, as required by a wet EEG system [23]. The electrodes used in dry EEG systems are commonly called dry electrodes because they are gel-free. They do not require conductive paste to establish contact with the scalp. These electrodes are often sold with a matching EEG headset or headcap with a 10–20 electrode arrangement. Dry EEG electrodes are categorized as passive or active electrodes. Dry EEG electrodes generally tend to have high electrode-to-skin contact impedance (typically between 100 and 2000 kΩ) [24,25]. Preamplification is required to prevent noise from contaminating weak EEG signals passing through the connector wires [26]. Active dry electrodes contain in-built preamplification circuitry or are paired with such circuitry, which is usually located at the connector portion closest to the electrode body. 

Commercially available dry EEG electrodes are typically made of metal alloy material, flexible polymer material, or a combination of these materials [27]. Moreover, hybrid electrode systems that contain dry EEG electrodes and other types of electrodes have been developed. For example, dry EEG electrodes have been combined with transcranial direct-current stimulation electrodes in a single headset [28]. With regard to the electrode shape and structure, electrodes made of stainless steel have a flat and round structure, whereas those made of gold alloy have a single-pin-based or multiple-pin-based structure that is fully rigid, spring-loaded, or placed in a rotating holder [29,30]. The use of metal pins leaves impressions on the skin and causes discomfort to patients when EEG signal recording is conducted for an extended period. Dry electrodes are made of flexible materials such as nylon, which is 3D printed onto a multilegged structure and coated with conductive polymer [31]. The problem of coating a conductive layer on a structure is that this coating is that it is prone to peeling off after repeated use, which can lead to degradation in impedance readings.

In the present study, passive dry EEG electrodes with an impedance of less than 150 kΩ were developed. The novelty of the proposed electrodes is the hybrid packaging of polydimethylsiloxane (PDMS) with a partially embedded polyimide-based flexible printed circuit board (FPCB). The electrodes are flexible and deformable with low contact impedance against the scalp. Scalp preparation using the proposed electrode is not necessary, which simplified the setup process and improve user experience, reduce discomfort as compared to wet electrode system. The electrodes are compatible with any EEG system that has a standard-sized snap connector cable and a Deutsches Institut für Normung plug connector. To obtain better EEG signal quality, the electrodes can be easily converted into active electrodes via pre-amplification circuitry. A single-piece fully adjustable and stretchable head cap with a built-in harness was fabricated for the electrodes according to the 10–20 arrangement during signal measurements. The development of the dry EEG electrodes and the EEG cap was previously published in 2020 [32]. This publication presented the development of the dry EEG head cap. For this work, we deep dive into dry electrode designs and their fabrication method. In addition, extensive work on electrode impedance testing using various types and combination of electrodes was conducted. The active dry EEG electrodes were further integrated with a circuitry module and tested on human subjects for brain signal recording. Data were transmitted to a mobile APP for signal readout. The results are discussed in this paper. 

## 2. Materials and Methods

**Figure 1:**
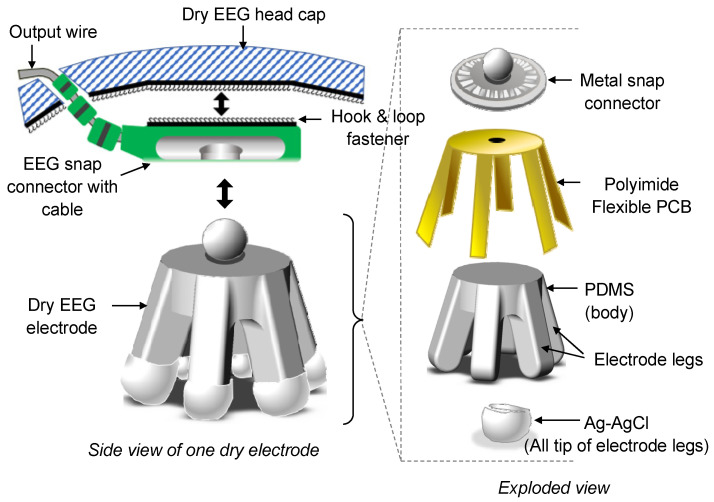
Designed dry electroencephalogram (EEG) electrode and its corresponding snap connector in the designed dry EEG head cap (not drawn to scale). The exploded view shows the configuration of the designed electrode, which consists of a rigid metal snap connector, partially embedded polyimide flexible printed circuit board (FPCB), polydimethylsiloxane, and tips coated with Ag–AgCl.

Two types of dry electrodes were developed: normal-legged and flat-disk electrodes. Normal-legged electrodes can generally be used on any part of the head with thick hair, whereas flat-disk electrodes can only be used on the portion of the head with no hair or limited hair (e.g., forehead). The design of the fabricated electrodes is displayed in Figure 1. The developed dry EEG electrodes are included in a system that also contains a dry EEG head cap and EEG cables. These electrodes contain a rigid metal snap connector, a polyimide FPCB, PDMS, and Ag–AgCl. The Ag–AgCl coating, which is biocompatible [33], is added to the electrodes to maximize the signal-to-noise ratio and stabilize the impedance of the electrodes. The dimensions of the rigid metal snap connector of the dry electrodes are compatible with those of the connector of the Holter electrocardiogram (ECG) cables used in this study. These cables are shielded and ready to use. Moreover, their end connectors are compatible with EEG equipment, such as the Nihon Kohden EEG system. 

**Figure 2:**
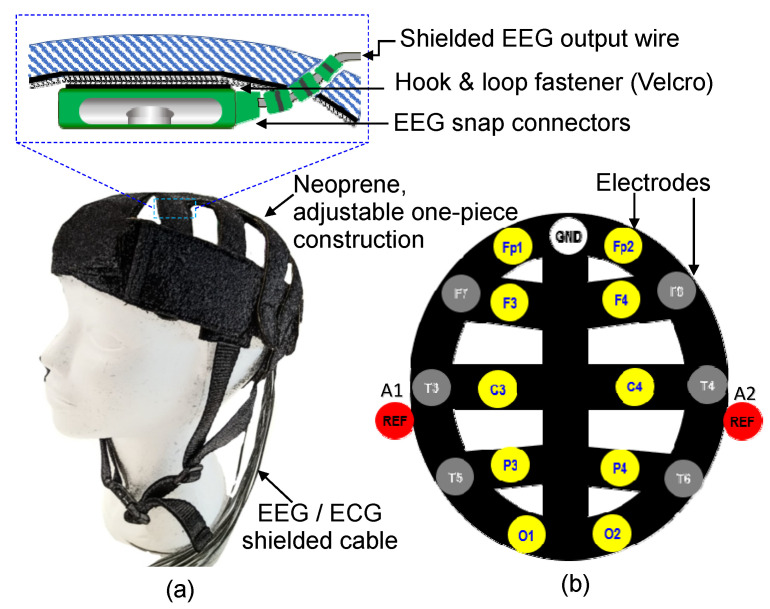
Design of the proposed dry EEG head cap: (**a**) shielded EEG cables attached with Velcro and fastened at the inner side of the cap. (**b**) Electrode arrangement (the 10–20 arrangement) and channels (Fp1, Fp2, F3, F4, C3, C4, P3, P4, O1, and O2) of the proposed head cap.

Figure 2 displays the design of the dry EEG head cap developed in this study. The designed cap can be adjusted according to the user’s head circumference. The snap connectors of the shielded EEG cables are attached to hook and loop fasteners, as shown in Figure 2a, for the positioning of the electrodes in the head cap. As displayed in Figure 2b, the developed head cap follows the 10–20 standard arrangement for electrode placement and is mainly used for signal measurement in the Fp1, Fp2, F3, F4, C3, C4, P3, P4, O1, and O2 channels. Ground and reference electrodes are also incorporated into the developed cap. Up to 19 channels can be used by incorporating additional EEG cables into the cap.

## 3. Fabrication and Packaging

### 3.1. Fabrication of Dry EEG Electrodes

Before the fabrication of dry EEG electrodes, EEG mold tools were designed and fabricated. The mold tools were designed according to the dimensions and geometry of the dry electrodes. The size of each electrode was primarily based on the 10–20 electrode arrangement for a head cap size of 46–52 cm for children and 60 cm for adults. In this study, the maximum head circumference for children was considered to be 54 mm. Table 1 lists the dimensions of the legged and flat-disk dry electrodes developed in this study. The protruding part of a flat-disk electrode that touches the skin is called a bump, which is equivalent to a leg of a normal-legged electrode. 

**Figure 3:**
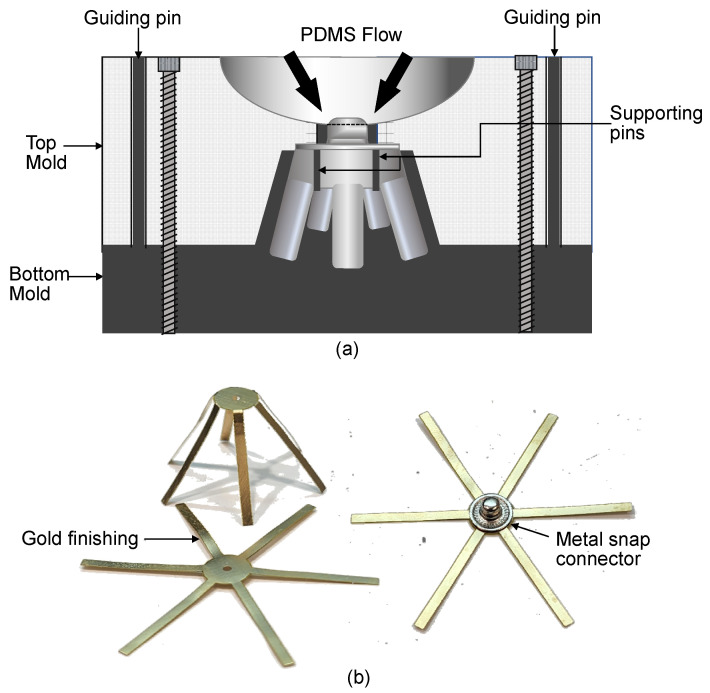
Photos of the mold tools and FPCB. (**a**,**b**) Mold tool of electrode design A. Each mold tool consists of a top and bottom mold, and FPCB which consists of a gold layer over a copper layer.

The mold tools for the electrodes were made from aluminum. Precision machining was adopted to create the shape of the designed electrode in the mold. Aluminum was used as the mold material because its high thermal conductivity allows PDMS to be cured uniformly and because it can withstand a curing temperature of 150 °C. As displayed in Figure 3a, the mold tools contained a top mold and bottom mold. Separate mold tools were developed for the legged (design A). To fix the metal snap connectors and FPCB in the correct positions, supporting pins were added to the bottom mold. The bottom mold also contained guiding pins for alignment with the top mold. When closed, the mold tools were secured by stainless-steel screws. For the easy release of cured PDMS, the mold tools were cleaned and conditioned after each curing process. A thin coating of conditioner was applied on the mold tools to prevent PDMS from sticking to the tools.

The main sensing element of the dry electrodes was the partially embedded FPCB. This board was fabricated with a polyimide and contained a 0.05 µm thick gold layer over a 34.7 µm thick copper layer. As shown in Figure 3b, the FPCB was designed as a common component for the two types of fabricated dry electrodes. The maximum thickness of the FPCB was 100 µm. The thinner the FPCB, the more flexible was the dry electrode when pressed down. Moreover, the thicker the FPCB was, the harder the dry electrode became. A thick FPCB has a thick polyimide or copper layer (>34.7 µm); this made it difficult for the FPCB to bend for the molding and casting of the electrode. A thicker copper layer increased the possibility of the FPCB containing a broken metal layer when bent; such a layer results in unstable or open impedance. The thickness of the copper layer in the FPCB was not greater than 34.7 µm, and the nominal polyimide thickness was maintained at 50 µm. 

The fabrication of a dry EEG electrode began with the soldering of a metal snap connector to an FPCB. A rigid metal snap connector was selected according to the usage of the standard ECG Holter cable connector. This connector is electrically conductive, has a typical resistance of <0.5 Ω, and is a solderable material. The soldering process was conducted using a hot plate at a temperature of 180 °C. The FPCB was laid flat on the hot plate, and a solder wire or solder paste was melted onto this plate. The metal snap connector was then placed directly onto the melted solder. The FPCB and snap connector were soldered together when they were lifted off the hot plate; the change in temperature hardened the melted solder. To verify this fusion, a continuity test was conducted using a digital multimeter. The soldering process could be repeated by placing the soldered part back on the hot plate. Figure 3b shows the metal snap connector soldered on the FPCB.

After soldering, the FPCB was placed inside a mold tool so that the FPCB could be preformed into the shape of the electrode. When the top mold was removed, the FPCB remained in the bottom mold, and the tips of the legs of the FPCB curled upward. The legs of the FPCB had to be inspected before the molding of PDMS. A 3 mm rigid tube was used for manually preforming the shape of the tips. This method was especially useful for the flat-disk electrodes. After the FPCB was suitably preformed and placed on the bottom mold, the top mold was placed back into the mold tool. The guiding pins of the bottom mold had to align with the holes of the top mold. Stainless-steel screws with a size of M4 were used to close the mold tool tightly.

Sylgard 160 was the PDMS selected for the body of the electrode. Sylgard 160 is a two-component silicone elastomer with a mixing ratio of 1:1 by weight or volume. This elastomer is curable at room temperature and elevated temperatures. When cured, PDMS has a dark gray color [34]. In this study, the curing temperature was set to 150 °C, and the curing time was set to 10 min. These settings were selected achieve a short curing time at elevated curing temperatures [35]. To remove trapped air or bubbles, the PDMS was deaired by using a vacuum jar at 40 mmHg for at least 30 min. After deairing was conducted, the PDMS was slowly poured into the cavity of the mold tool and allowed to rest for 15 min. The resting period facilitated the further deairing of the PDMS in the mold. Subsequently, conventional oven curing was performed, and the mold tool was allowed to cool down after this process. The stainless-steel screws and top mold were removed after the mold tool cooled down, and the electrode was separated from the bottom mold. For the legged electrodes, each leg was slowly released from the mold. After all the legs were released, visual inspection was conducted. Fine film-like protrusions from the electrode structure might occur if the top and bottom molds have a micro-gap during closure, which can be removed using a micro-grinder under a low-power microscope.

After the aforementioned steps, the FPCB was fully embedded. All the electrode legs (for legged electrodes) or bumps (for the flat-disk electrodes) were molded in PDMS. The metal layer of the FPCB at the tips of electrode legs and bumps had to be exposed to create an electrical connection. A micro-grinder was used to expose this layer partially, and a continuity test was then conducted using a digital multimeter. After the continuity was verified, the electrode was coated with Ag–AgCl. Ag–AgCl (ALS Co. Ltd., Osaka, Japan) has a surface resistance of 7.87 m Ω/µm^2^ and a viscosity of 50,000 ± 10,000 CP at 21.1 °C, and it can be used without further processing. This material can be applied to the intended surface through painting or dipping and is cured through air drying at room temperature or oven curing. To coat the electrode legs and bumps uniformly, Ag–AgCl was applied through the dipping process in this study. This process was performed by placing Ag–AgCl into a Petri dish and then dipping each electrode upright into the Petri dish up to 3 mm from its bottom. After the dipping process, the electrodes were placed upside down in a stainless-steel carrier. Ag–AgCl was cured at 120 °C for 5 min. The measured electrical resistance of the cured parts ranged from 0.1 to 0.3 Ω. After the aforementioned process, the dry electrodes were ready for use and testing.

**Figure 4:**
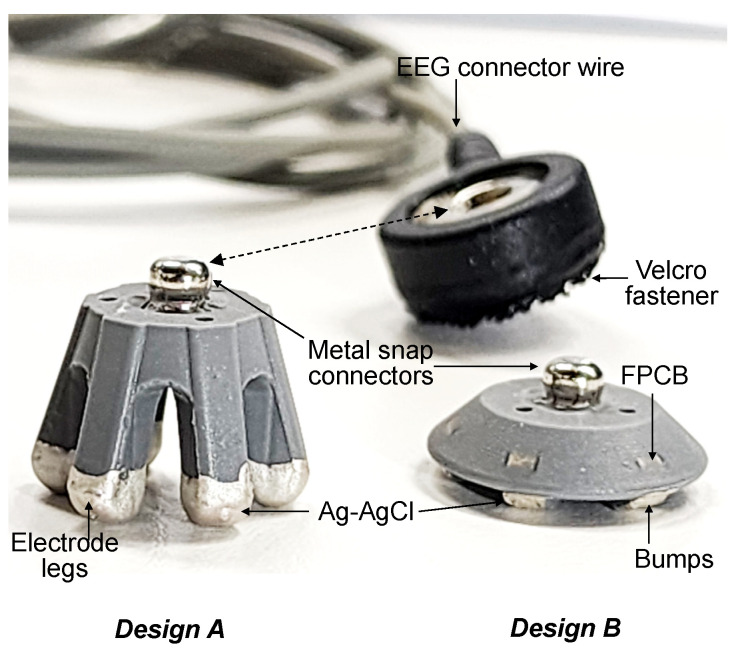
Photograph of the fabricated dry EEG electrodes. Design A is a normal electrode intended for use on hairy parts of the head, and design B is intended for use on portions of the head with limited hair. All electrode legs and bumps are coated with Ag–AgCl through dipping.

Figure 4 shows the fabricated legged and flat-disk EEG electrodes. The previously exposed metal layer of the polyimide FPCB was fully covered with cured Ag–AgCl. Although the metal snap connector was partially embedded in PDMS, this connector was still compatible with EEG cables. To avoid the discoloration or contamination of Ag–AgCl, the fabricated dry electrodes were placed in a clean container and stored inside a dry cabinet. The fabricated electrodes did not exhibit peeling of the coating. The main conductive layer was the FPCB, which was fully embedded in the electrode structure after the coating process. 

### 3.2. Fabrication of Dry EEG Head Caps

**Figure 5:**
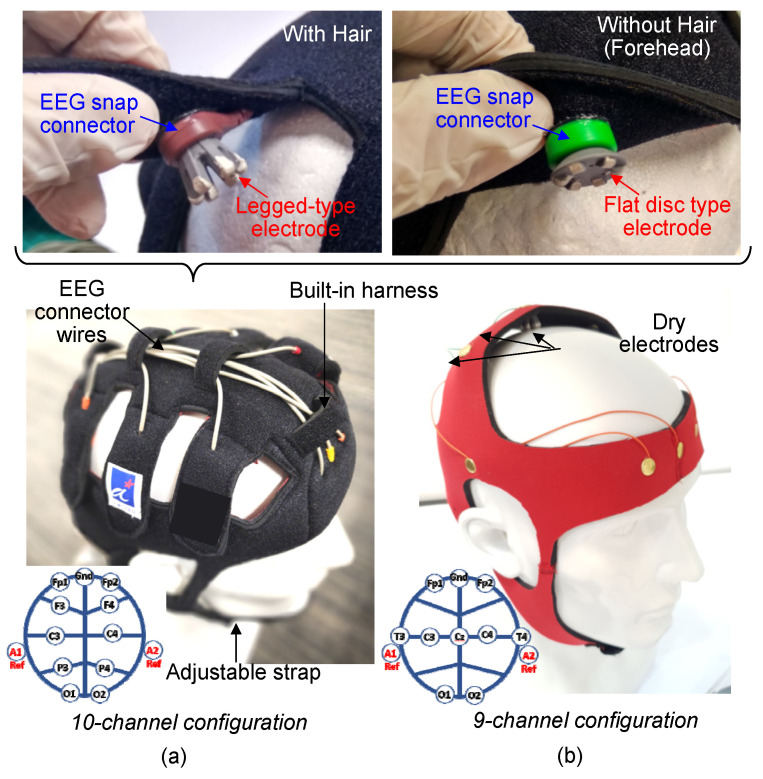
Photo of the fabricated dry head caps. The (**a**) 10-channel configuration and (**b**) nine-channel configuration follow the standard 10–20 electrode arrangement. Both one-piece structures can be integrated with EEG cables and allow the repositioning of dry EEG electrodes.

Dry EEG head caps were fabricated using a 3 mm thick neoprene fabric that is compatible with loop and hook fastener material (Velcro). The EEG connectors were attached with Velcro and were arranged on the head caps according to the standard 10–20 electrode arrangement. As displayed in Figure 5, two configurations of head caps were fabricated: a 10-channel head cap and 9-channel head cap. The 10-channel head cap can optionally hold additional electrode cables for up to 19 channels. This cap was fabricated with maximum sizes of 52 and 60 cm for children and adults, respectively. A ground electrode and two reference electrodes are incorporated in the aforementioned head cap. The ground electrode is located at the forehead between the two prefrontal electrodes (Fp1 and Fp2), and one reference electrode is located behind each ear. EEG cables are repositioned or added through the built-in hardness of the head cap. The inner part of this cap is lined with water-resistant fabric that can be cleaned with disinfectant wipes after each use. The fabricated 10-channel head cap has adjustable webbing straps and a chin guard.

The nine-channel head cap was fabricated for use on the motor cortex area of the head. The channels corresponding to this area are Fp1, Fp2, T3, T4, C3, Cz, C4, O1, and O2. The nine-channel head cap incorporates most of the features of the 10-channel one except for the adjustable straps. The nine-channel head cap was fabricated from stretchable neoprene material. In contrast to the 10-channel head cap, the nine-channel head cap has a fixed number of channels with no option to add additional EEG cables.

## 4. Test Results and Discussion

### 4.1. Impedance Test

#### 4.1.1. Impedance Readings Obtained Using the Nihon Kohden EEG System

**Figure 6:**
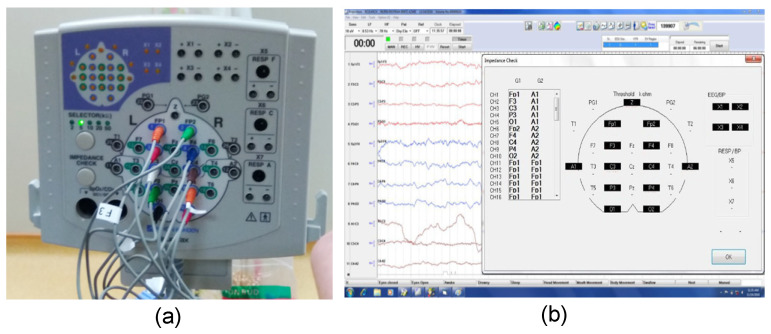
Photographs of (**a**) the Nihon Kohden junction box with EEG cables plugged into the target electrode locations. (**b**) Impedance values shown on the display of the Nihon Kohden EEG system.

Impedance testing was conducted using the Nihon Kohden EEG system on five flat-disk and eight legged dry EEG electrodes. The fabricated 10-channel EEG head cap was used for impedance testing. The EEG cables attached to this head cap were connected to the junction box of the Nihon Kohden EEG system (Figure 6a). Conventional gold disk electrodes with conductive paste were also prepared to obtain typical impedance values for a wet EEG system. After the impedance readings were checked, participants were asked to open and close their eyes to examine whether the fabricated dry electrodes could detect EEG signals when paired with a Nihon Kohden junction box. The adopted test protocol involved the following steps:

1. Testing was performed using only dry EEG electrodes attached to the fabricated 10-channel head cap. The ground electrode (Z) and reference electrodes (A1 and A2) were also dry electrodes.
2. Testing was performed again by replacing five dry electrodes, namely A1, A2, C3, C4, and Z, with wet electrodes.
3. Testing was reperformed using only conventional wet electrodes.

The aforementioned protocol was followed to evaluate the entire dry system without altering the condition of the target area. The wet electrode setup requires the scalp preparation and application of conductive paste on the electrode during placement. Table 2 presents the impedance levels measured by the EEG system. These values were displayed on the liquid crystal display monitor of the EEG system, as shown in Figure 6b. The impedance values of the full dry system and the mixed system with eight dry and five wet electrodes were <65 kΩ. However, the full wet system exhibited impedance values of less than 5 kΩ, mainly because of the skin preparation and the conductive paste on each electrode. 

#### 4.1.2. Impedance Readings Obtained Using the NuAmps Amplifier

**Figure 7:**
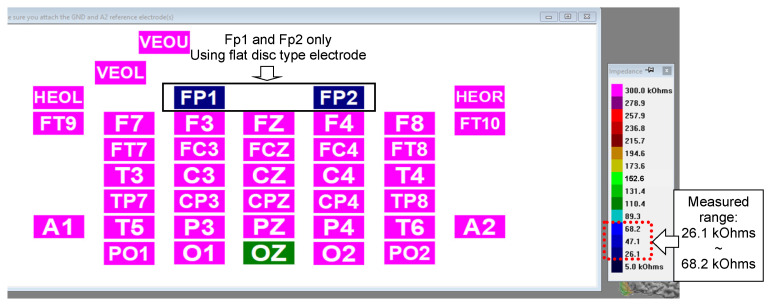
Photograph of the display screen of the NuAmps amplifier when measuring the impedance of flat-disk electrodes. The value of the measured impedance is indicated by the color scale to the right. All the electrodes for which values are displayed in magenta were not connected.

The impedance levels of the flat-disk electrodes were also measured using the NuAmps amplifier (Compumedics Neuroscan) to examine whether these electrodes could measure impedance when paired with the NuAmps amplifier. The electrodes were attached to the Fp1 and Fp2 locations of the head cap, and the remaining electrode locations were not connected because they required legged electrodes. The impedance values of the unconnected electrodes were shown in magenta on the display of NuAmps. As displayed in Figure 7, the impedance values of the flat-disk electrodes were approximately 26.1–68.3 kΩ. The maximum impedance reading was within the impedance range obtained with the Nihon Kohden system.

### 4.2. EEG Signal Recording

#### 4.2.1. Verification of the Pairing of the Fabricated Dry EEG Electrodes

**Figure 8:**
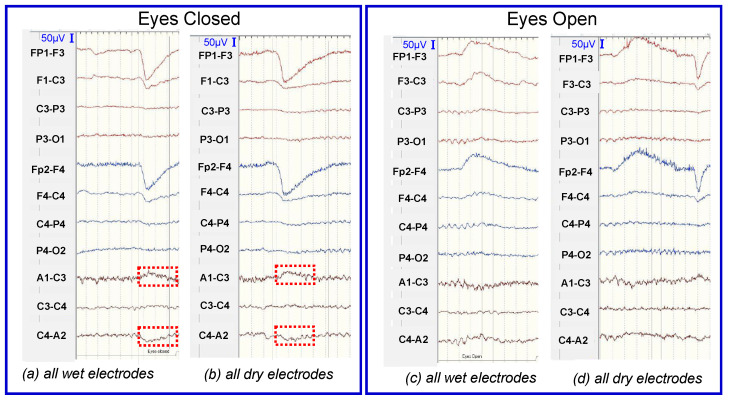
Brain signals recorded by using Nihon Kohden system: signals obtained when using (**a**) the full wet electrode system under the eyes closed condition, (**b**) the full dry electrode system under the eyes closed condition, (**c**) the full wet electrode system under the eyes open condition, (**d**) the full dry electrode system under the eyes open condition.

In Figure 8, brain signals were recorded using the Nihon Kohden EEG system to investigate whether the EEG signals obtained with the dry electrodes were as consistent as those obtained using conventional wet electrodes. The EEG equipment setting for low pass filter is 0.53 Hz, high pass filter is 70 Hz, and the sensitivity is 10 µV. The recording was targeted in low frequency domain, purposely for epilepsy monitoring. The EEG equipment also has a high signal-to-noise-ratio which filters out background noise to obtain the usable signal. Participants were asked to open and close their eyes to examine whether the changes in the brain wave pattern were detected by the electrodes. 

One-way analysis of variance (ANOVA) in OriginPro software (OriginLab Corporation, Northampton, MA, USA) was used to perform statistical analysis on the measured brain wave data. One-way ANOVA compared the mean amplitude of wet and dry electrodes for statistically significant difference [36]. When ANOVA is performed, results produced corresponding F-Value and *p*-level. F-value is the variation between samples, thus the larger it is, the greater the difference between the means. The *p*-level determines if the difference between means is statistically significant, i.e., if *p* is less than 0.05 [37], there is a significant difference between mean amplitudes of the signals between the wet and dry electrodes. The electrodes from placement A1–C3 and at C4–A2 are selected for analysis. Signal used for the analysis is shown in Figure 8a,b. The results of the ANOVA showed that there was no statistically significant difference in amplitudes between wet and dry electrodes at placement A1–C3 (F-value = 0.5787, *p* = 0.4499) and C4–A2 (F-value = 0.3284, *p* = 0.5688). Figure 8 indicates that despite the big difference in the electrode impedance levels, the amplitudes of the signals recorded from dry electrodes are comparable to those recorded obtained using conventional wet electrodes. 

#### 4.2.2. Capturing EEG Signals by Using Self-Developed Circuitry

**Figure 9:**
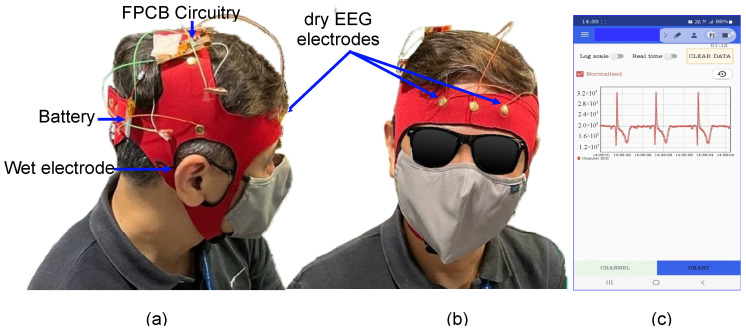
Photographs of the setup comprising a mobile application (app) and a flexible circuitry board. (**a**) Two flat-disk electrodes placed at Fp1 and Fp2. The flexible circuitry is placed as close as possible to these electrodes, (**b**) wet reference electrode placed behind the ear, and (**c**) mobile app used to read EEG signals through 1000× gain amplification.

Further assessment of the fabricated dry electrodes was conducted with and without the 10-channel head cap to verify whether these electrodes could capture signals that were similar to signals in the alpha bandwidth. A mobile application (app) was developed to record and measure brain signals and used in the aforementioned assessment. This app was paired with an FPCB, to which the fabricated dry electrodes were connected. Two flat-disk electrodes were placed on the forehead (at Fp1 and Fp2). Moreover, a wet Au disk electrode with Ten20 conductive paste was placed behind the right ear at A2. This electrode served as the reference electrode. The developed flexible circuitry only allows for the connection of two EEG electrodes and one reference electrode, and this circuitry is placed as close as possible to the two EEG electrodes. Figure 9 displays the setup used in the aforementioned assessment.

The bandwidth of the amplifier was set to 40 Hz. The flexible circuit board’s input alternating current had a frequency of 200 Hz, and the amplification gain was set as 1000×. The resolution of the embedded analog-to-digital converter was set as 1 mV, and the Bluetooth module of the system logged real-time data.

**Figure 10:**
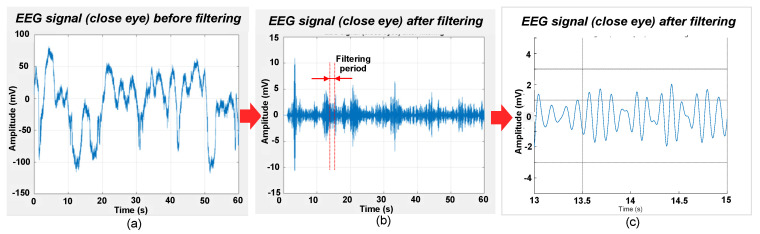
(**a**) EEG signal acquired before filtering, (**b**) after filtering within a bandwidth of 8–12 Hz, and (**c**) signal zoomed-in to a period of 13–15 s from (**b**).

The adopted protocol involved participants engaging in the following sequence: eyes closed for 60 s, eyes open for 60 s, and blinking for 60 s. This protocol was repeated twice for each participant, and corresponding data were collected. The peak-to-peak amplitude of this signal was 4 mV; thus, the actual peak-to-peak amplitude was 4 μV because the amplification gain was 1000 times. The mobile app recorded all the data generated during the testing, which were then exported for postprocessing. The acquired signals were postprocessed and filtered to a bandwidth of 8–12 Hz. Filtering was done to eliminate the out-of-band noise to obtain alpha-band EEG signal [38]. Digital filter design called FDA Tool in MATLAB (The MathWorks Inc., Natick, MA, USA) was employed to implement a bandpass filter for the signal processing. Chebyshev type filter was chosen due to its sharp transition between passband and stopband with a lower order. The passband was 8~12 Hz under sampling frequency of 200 Hz. The passband and stopband attenuation were both set to be 80 dB with passband ripple of 0.01 dB, which was adequate for signal quality. Figure 10a,b show raw EEG signal before and after filtering, respectively. After bandpass filtering, a reduction in amplitude from ±100 mV to ±10 mV, which is more than ten times (10×), was observed. Figure 10c shows that when zooming-in to a period of 13–15 s, the EEG signal waveforms are observed to be similar to the alpha signals from brainwave [38]. After the amplification gain was set as 1000 times, the flexible circuit board captured EEG signals and wirelessly display signals in the developed mobile app. 

### 4.3. Primary Skin Irritation Tests and Biocompatibility Tests

**Figure 11:**
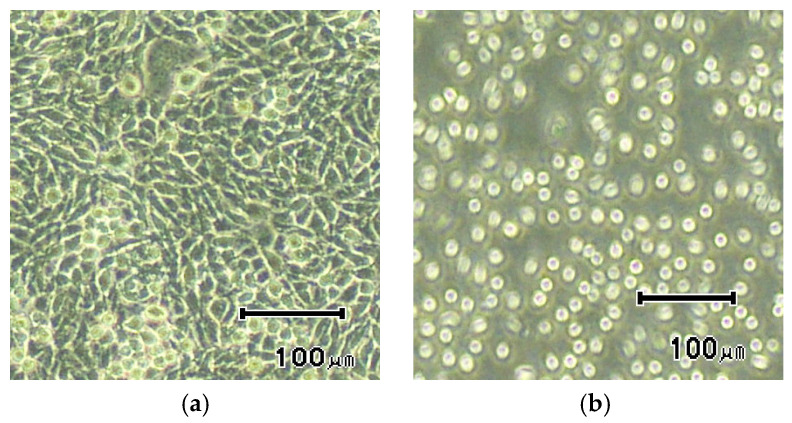
Photographs of cytotoxicity result (**a**) test sample, (**b**) positive control.

The fabricated dry EEG electrodes were tested according to the ISO 10993 protocol, which describes tests for skin irritation and delayed hypersensitivity. In the ISO 10993 tests, the fabricated dry EEG electrodes exhibited no adverse effect on all the tested animals during the observation period. To examine the biocompatibility of these electrodes, cytotoxicity tests based on the ISO 10993 protocol, which are tests for in vitro cytotoxicity, were performed. The results of these tests (Figure 11) indicated that the fabricated dry EEG electrodes had no cytotoxic effect. Thus, the fabricated dry EEG electrodes are biocompatible.

## 5. Conclusions

In this study, we fabricated flexible dry EEG electrodes with impedance values of <150 kΩ. Partially embedding the polyimide flexible PCB in PDMS and Ag–AgCl coating at the tip of the electrode legs enabled a steady low-contact-impedance readings. There is no peeling-off of conductive layer observed from the electrode body after multiple usages. The electrodes passed the test for primary skin irritation and biocompatibility.

Universal single-piece and stretchable dry EEG head caps were also developed. The use of Velcro-compatible neoprene fabric caters to easy repositioning of the electrodes while still following the 10–20 standard placement. Additional EEG connector wires are also possible through the built-in harness of the head cap. The dry EEG head cap sizes fit head circumferences of 46–52 cm for children and 60 cm for adults. The brain signals detected by the fabricated dry EEG electrodes are similar to those detected by wet electrodes. The fabricated dry EEG electrodes are compatible with hospital-grade EEG amplifiers and with a self-developed system featuring flexible circuitry and a mobile application. EEG signal acquired from human subjects had been amplified at a gain of 1000 times. Post processed EEG signal waveforms were transmitted wirelessly to the developed mobile application for display. This study is useful especially in areas where extended recording of EEG is needed. The developed dry electrode can be paired with various types of head caps and dry EEG systems for clinical setting, non-laboratory setting, or even in gaming. It can be further developed for use in neuroscience or neuromonitoring applications such as upper limb rehabilitation and EEG signal acquisition for potential customer preference in fragrance and flavor marketing. 

## Figures and Tables

**Table 1:** Comparison of the legged and flat-disk dry EEG electrodes developed in this study.

Description	Design A (Legged)	Design B (Disc Type)
Span (as is)	16.0 mm	16.0 mm
Total Thickness ^1^	13.6 mm	4.5 mm
Span ^2^	22.0 mm	16.0 mm
No. of Legs/Bumps	6 legs	6 bumps
Leg Length	7.0 mm	N/A
Leg Thickness	3.5 mm	N/A
Leg/Bump Width	3.0 mm	3.0 mm
Ag–AgCl on Tip/Bumps	Yes	Yes

^1^ Total thickness includes the soldered metal snap connector. ^2^ The span of each electrode is from one of its tips to the other when the electrode is flattened or pressed down.

**Table 2:** Impedance levels recorded using the Nihon Kohden junction box.

Electrode	Dry Electrodes (kΩ)	Dry (8 Electrodes) + Wet (A1, A2, C3, C4, Z) Electrodes (kΩ)	Wet Electrodes ^1^ (kΩ)
Z	50.52	2.46	2.88
Fp1	58.60	17.79	4.79
F3	56.30	56.41	4.53
C3	13.68	2.48	1.23
P3	57.63	11.74	2.57
O1	57.51	8.00	4.81
Fp2	60.57	59.50	4.68
F4	63.84	63.84	4.08
C4	11.79	2.42	1.19
P4	60.67	22.80	2.26
O2	60.44	12.98	3.61
A1	60.56	1.68	2.07
A2	61.09	1.52	1.55

^1^ Typical impedance values of the wet electrode when using an Au cup electrode.

## Data Availability

https://www.a-star.edu.sg/ime (accessed on 29 April 2023).
